# Supplementary material for: Cannabinoid Receptor 2 Alters Social Memory and Microglial Activity in an Age-Dependent Manner
Source: Molecules. 2021 Oct 2;26(19):5984. doi: 10.3390/molecules26195984 (PMC8513097; doi:10.3390/molecules26195984)
Supplement: Supplementary file 1 [file molecules-26-05984-s001.zip › molecules-1346279-Supplementary.pdf]

Supplementary Material

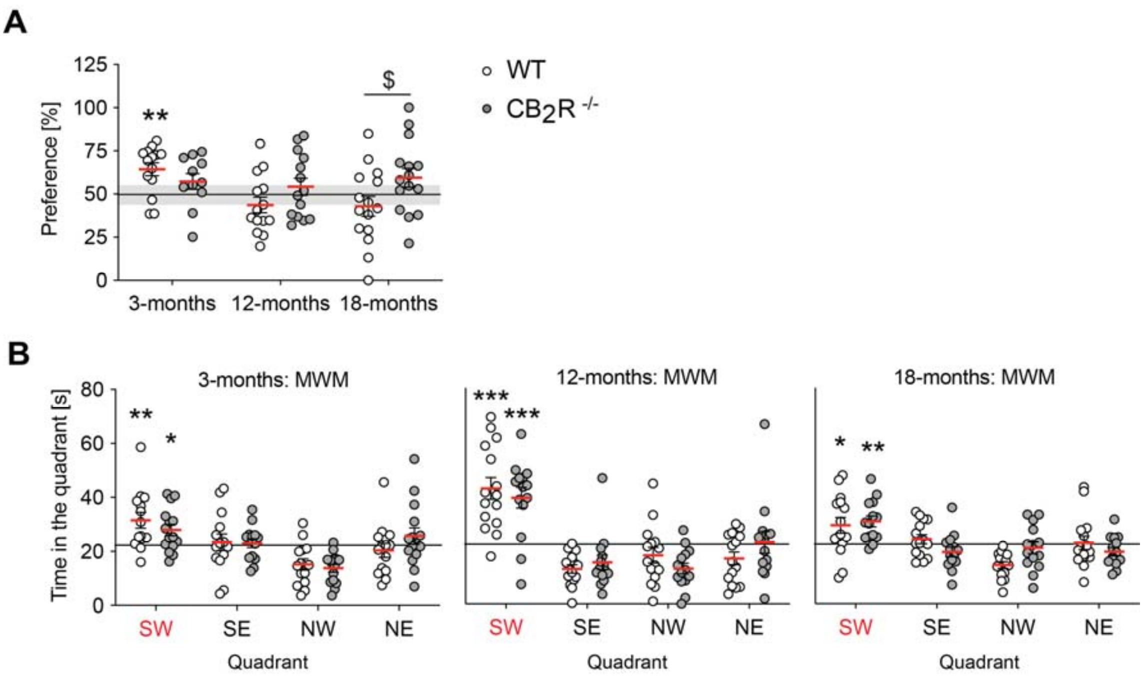

Supplementary Figure S1: Memory (**A**) Preference for the novel partner (after 30 minutes separation) was calculated as time with the novel partner mouse over total interaction time. Each group was analysed individually by one-sample t-test (hypothetical mean = 50). Significant difference from the 50 % chance level indicated learning with $^{**}p< 0.01$. Each point represents a single mouse. Red line indicates the mean value ± SEM. Line indicates a 50 % chance level. Grey box indicates 5 % variance around the chance level. Two-way ANOVA followed by Sidak's multiple comparison test with $ $p< 0.05$ significance between genotypes within the same age group. (**B**) Probe trial of the MWM. Panels from left to right: 3-, 12- and 18-months old mice. Each group was analysed individually by one-sample t-test (hypothetical mean = 22.5). Line indicates a 22.5 s chance level with total swim time 90 s. Quadrant in which the platform was localized (SW) indicated in red. SW - southwest; SE - southeast; NW - northwest; NE - northeast. Significant difference from the 22.5 s chance level indicated intact memory recall with $p< 0.05$; $^{**}p< 0.01$; $^{***}p< 0.001$

WT mice – white circle; $CB_2R^{-/-}$ mice – grey circles. N=14-15 mice / genotype / age group.

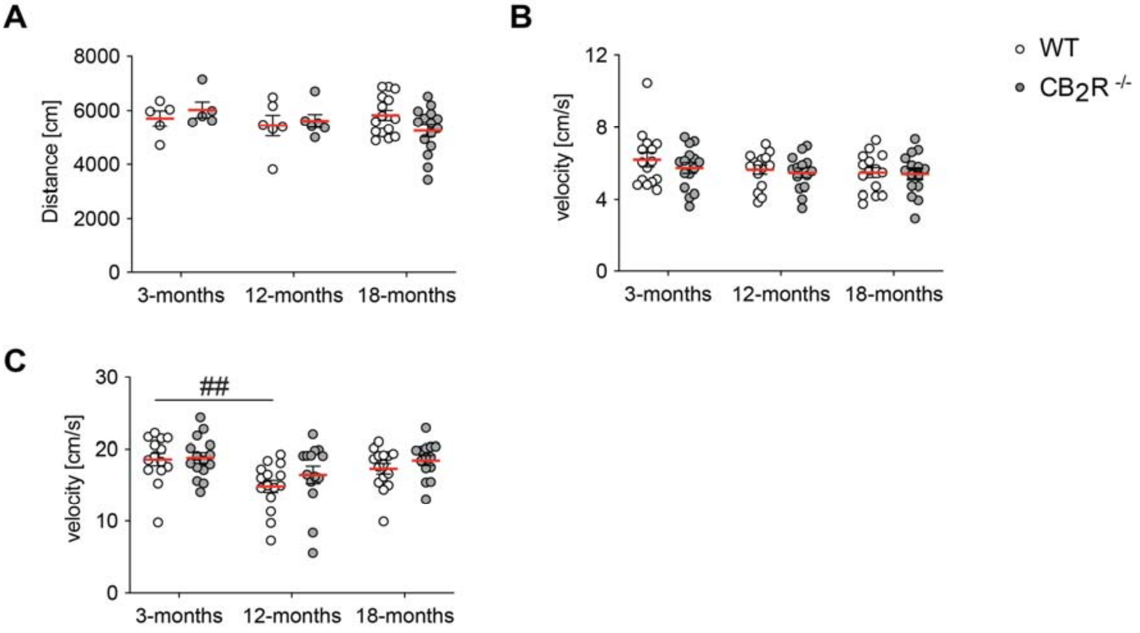

Supplementary Figure S2: Motility (**A**) Total distance travelled during open-filed like test (habituation of the PR test, 10-minute free exploration of the open-field box). (**B**) Mice velocity during trial 1 of the PR test. (**C**) Mice velocity during probe trail of the MWM test. Two-way ANOVA followed by Sidak's multiple comparison test with ##*p*< 0.01 significance between age groups within the same genotype. WT mice – white circle; $CB_2R^{-/-}$ mice – grey circles. N=5-15 mice / genotype / age group.

Supplementary Table S1. Statistical analysis.

		Statistical test		Posthoc test	
**Figure**	**Panel**	**Test name**	**result**	**Test name**	**result**
**1. CB2R deletion has a moderate age-dependent effect on cognition.**	**1b:** Sociability (30 min)	one-sample t-test (hypothetical mean = 50)	**all groups** p< 0.0001		
		two-way ANOVA	Interaction age x genotype F (2, 71) = 0.8353, p = 0.4379; age F (2, 71) = 1.018, p = 0.3664; genotype F (1, 71) = 1.041, p = 0.3111		
	**1c:** Novelty preference (30 min)	one-sample t-test (hypothetical mean = 50)	**3-month WT** p =0.0468, CB2R-/- p = 0.9620; **12-month WT** p = 0.0035, CB2R-/- p = 0.0906; **18-month WT** p =0.7849, CB2R-/- p = 0.0472		
		two-way ANOVA	age x genotype F(2, 71) = 3.560; p = 0.0336; age F (2, 71) = 1.136, p = 0.3270; genotype F (1, 71) = 0.7894, p = 0.3773	Šídák's multiple comparisons test	**WT** 3-month vs 18-month p = 0.0391
	**1d:** 3-month old (Acquisition)	two-way RM Anova	interaction time x genotype F(5, 135) = 0.6925; p = 0.6300; time F(5, 135) = 12.29; p< 0.0001; genotype F(1, 27) = 0.0006; p = 0.9800	Šídák's multiple comparisons test	**WT** Day 1 vs Day 4 p = 0.0245; Day 1 vs Day 6 p = 0.0010; Day 2 vs Day 4 p = 0.0427; Day 2 vs Day 6 p = 0.0019; **CB2R-/-** Day 1 vs Day 4 p = 0.0047; Day 1 vs Day 5 p = 0.0001; Day 1 vs Day 6 p < 0.0001; Day 2 vs Day 6 p = 0.0213
	**1d:** 3-month old (Reversal)	two-way RM Anova	interaction time x genotype F(2, 54) = 0.711; p = 0.4957; time F(2, 54) = 8.366; p= 0.0007; genotype (F1, 27) = 0.1297; p = 0.7215	Šídák's multiple comparisons test	**CB2R-/-** Day 8 vs Day 9 p = 0.0124; Day 8 vs Day 10 p = 0.0024
	**1d:** 12-month old (Acquisition)	two-way RM Anova	interaction time x genotype F(5, 135) = 2.06; p = 0.0743; time F(5,135) = 33.06; p< 0.0001; genotype F(1, 27)= 0.0017; p = 0.8950	Šídák's multiple comparisons test	**WT** Day 1 vs Day 2/3/4/5/6 p < 0.0001; Day 2 vs Day 5 p = 0.0255; Day 2 vs Day 6 p = 0.420; **CB2R-/-** Day 1 vs Day 3 p = 0.0027; Day 1 vs Day 4/5/6 p < 0.0001; Day 2 vs Day 3 p = 0.0269; Day 2 vs Day 4 p = 0.0006; Day 2 vs Day 5/6 p < 0.0001; Day 3 vs Day 6 p = 0.0310
	**1d:** 12-month old (Reversal)	two-way RM Anova	interaction time x genotype F(2, 54) = 3.92; p = 0.0257; time F(2, 54) = 20.04; p< 0.0001; genotype F(1,27)= 1.299; p = 0.2644	Šídák's multiple comparisons test	**Day 9 WT vs CB2R-/-** p = 0.0610; **WT** Day 8 vs Day 9/10 p < 0.0001; **CB2R-/-** Day 8 vs Day 10 p= 0.0072; Day 9 vs Day 10 p= 0.0402

		Statistical test		Posthoc test	
**Figure**	**Panel**	**Test name**	**result**	**Test name**	**result**
	**1d:** 18-month old (Acquisition)	two-way RM Anova	interaction time x genotype F(5, 135)= 1.081; p = 0.3739; time F(5,135) = 31.62; p< 0.0001; genotype F(1,27) = 0.907; p = 0.3494	Šídák's multiple comparisons test	**WT** Day 1 vs Day 2 p = 0.0080; Day 1 vs Day 3/4/5/6 p < 0.0001; Day 2 vs Day 5 p = 0.0412; Day 2 vs Day 6 p = 0.0003; **CB2R-/-** Day 1 vs Day 2/3/4/5/6 p< 0.0001
	**1d:** 18-month old (Reversal)	two-way RM Anova	interaction time x genotype F(2, 54) = 1.936; p = 0.1541; time F(2, 54) = 12.82; p < 0.0001; genotype F(1, 27) = 0.07016; p = 0.7931	Šídák's multiple comparisons test	**CB2R-/-** Day 8 vs Day 9 p= 0.0113, Day 8 vs Day 10 p< 0.0001
**2. CB2R deletion results in a decreased anxiety phenotype in o-maze**	**2a:** % of time spent in the open compartment	two-way ANOVA	Interaction age x genotype F (2, 82) = 0.3559, p = 0.7016; age F(2, 82) = 10.26; p = 0.0001; genotype F(1, 82) = 4.427; p = 0.0384	Šídák's multiple comparisons test	**WT** 3-months vs 18-months p= 0.0026; **CB2R-/-** 3-months vs 12-months p= 0.0372, 3-months vs 18-months p= 0.0306
	**2b:** Distance traveled in the open compartment	two-way ANOVA	Interaction age x genotype F(2, 82) = 0.5281, p= 0.5917; age F(2,82) = 17; p < 0.0001; genotypeF(1,82) = 7.009, p= 0.0097	Šídák's multiple comparisons test	**WT** 3-months vs 18-months p= 0.0004; **CB2R-/-** 3-months vs 12-months p= 0.0012, 3-months vs 18-months p= 0.0007
	**2c:** Number of stretched posture behaviours	two-way ANOVA	Interaction age x genotype F(2, 82) = 3.004, p = 0.0552, age F(2, 81) = 4.152; p= 0.0192; genotype F(1, 81) = 13.24; p = 0.0005	Šídák's multiple comparisons test	**WT vs CB2R-/- 3-months** p= 0.0071, **WT vs CB2R-/- 18-months** p= 0.0072; **CB2R-/-** 12-month vs 18-months p =0.0083
	**2d:** Number of looking down behaviors	two-way ANOVA	Interaction age x genotype F(2, 81) = 0.6890, p = 0.5050, age F(2, 81) = 2.702; p =0.0731, genotype F(1, 81) = 33.24; p < 0.0001	Šídák's multiple comparisons test	**WT vs CB2R-/- 3-months** p= 0.0039, **WT vs CB2R-/- 12-months** p= 0.0451; **WT vs CB2R-/- 18-months** p= 0.0002
**3. Accumulation of Lipofuscin in pyramidal neurons during aging is not altered by CB2 deletion**	**3b:** Area covered	two-way ANOVA	interaction, genotype x age F(2,66) = 0.4423; p = 0.6445; age F(2,66)= 81.41; p < 0.0001; genotypeF(1,66) = 0.5129; p = 0.4764	Šídák's multiple comparisons test	**WT** 3-months vs 12-months p = 0.0001, 3-months vs 18-months p < 0.0001, 12-months vs 18-months p < 0.0001; **CB2R-/-** 3-months vs 12-months p < 0.0001, 3-months vs 18-months p < 0.0001, 12-month vs 18-month p = 0.0034
	**3c:** Particle density	two-way ANOVA	interaction genotype x age F(2, 65) = 0.7953; p = 0.4558; age F(2, 65) = 82.98; p< 0.0001; genotype F(1, 65) = 1.269; p = 0.2641	Šídák's multiple comparisons test	WT 3-months vs 12-months p < 0.0001; 3-months vs 18-months p < 0.0001; 12-months vs 18-months p = 0.0010; CB2R-/- 3-months vs 12-months p < 0.0001; 3-months vs 18-months p<0.0001; 12-months vs 18-months p = 0.0527

		Statistical test		Posthoc test	
**Figure**	**Panel**	**Test name**	**result**	**Test name**	**result**
**4. Accumulation of Lipofuscin in microglia is increased after CB2 deletion**	**4b:** Area covered	two-way ANOVA	interaction genotype x age F(2, 419)= 7.514; p = 0.0006; age F(2, 419) = 47.06; p < 0.0001; genotype F(1,419) = 7.857; p = 0.0053	Šídák's multiple comparisons test	**WT vs CB2R-/- 12 months** p< 0.0001; **WT** 3-months vs 18-months p< 0.0001, 12-months vs 18-months p< 0.0001; **CB2R-/-** 3-months vs 12-months p< 0.0001, 3-months vs 18-months p<0.0001
	**4c:** Particle density	two-way ANOVA	interaction genotype x age F(2,429) = 1.085; p = 0.3388; age F(2,429) = 48.34; p< 0.0001; genotype F(1,419)= 3.209; p = 0.0740	Šídák's multiple comparisons test	**WT** 3-months vs 12-months p < 0.0001, 3-months vs 18-months p < 0.0001; **CB2R-/-** 3-months vs 12-months p< 0.0001; 3-months vs 18-months p<0.0001; 12-months vs 18-months p = 0.0385
**5. Iba1 and CD68 intensity is enhanced in CB2-/- microglia**	**5b:** Iba1 mean grey value	two-way ANOVA	interaction genotype x age F(2,345) = 3.525; p = 0.0305; age F(2,345) = 11.43; p < 0.0001; genotype F(1,345) = 36.48; p < 0.0001	Šídák's multiple comparisons test	**WT vs CB2R-/- 3 months** p < 0.0001; **WT vs CB2R-/- 18 months** p < 0.0001; **WT** 3-months vs 18-months p < 0.0001, 3-months vs 18-months p = 0.0465; 3-months vs 18-months p = 0.0511; **CB2R-/-** 3-months vs 18-months p =0.0373
	**5c:** % of soma covered by CD68	two-way ANOVA	interaction genotype x age F(2,334) = 2.424; p = 0.0901; age F(2,334) = 18.11; p< 0.0001; genotype F(1,334) = 2.794; p = 0.0956	Šídák's multiple comparisons test	**WT vs CB2R-/- 18 months** p = 0.0342; **WT** 3-months vs 12-months p < 0.0001, 3-months vs 18-months p = 0.0347; **CB2R-/-** 3-months vs 12-months p =0.0046; 3-months vs 18-months p < 0.0001
**6. Age-dependent alteration in expression of inflammatory mediators.**	**6a:** *tnfα* relative expression	two-way ANOVA	interaction genotype x age F(2,28) = 2.191; p = 0.1306; age F(2,28) = 7.804; p = 0.002; genotype F(1,28) = 3.673; p = 0.0656	Šídák's multiple comparisons test	**CB2R-/-** 3-month vs 12-month p = 0.0095, 3-month vs 18-month p = 0.0022
	**6b:** *il-6* relative expression	two-way ANOVA	interaction genotype x age F(2,28) = 1.152; p = 0.3304; age F(2,28) = 15.08; p< 0.0001; genotype F(1,28) = 1.089; p = 0.3057	Šídák's multiple comparisons test	**WT** 3-month vs 12-month p = 0.0025; 12-month vs 18-month p = 0.0052; **CB2R-/-** 3-month vs 12-month p = 0.0018
	**6a:** *ccl2* relative expression	two-way ANOVA	interaction genotype x age F(2,29) = 4.738; p = 0.0166; age F(2,29) = 12.29; p = 0.0001; genotype F(1,29) = 0.5880; p = 0.4494	Šídák's multiple comparisons test	**WT vs CB2R-/-** p = 0.0266; **WT** 3-month vs 18-month p < 0.0001, 12-month vs 18-month p = 0.0001
	**6b:** *arg1* relative expression	two-way ANOVA	interaction genotype x age F(2,30) = 0.3524; p = 0.7059; age F(2,28) = 1.803; p = 0.1823; genotype F(1,30) = 0.0531; p = 0.8193		

		Statistical test		Posthoc test	
**Figure**	**Panel**	**Test name**	**result**	**Test name**	**result**
	**6b:** *nos2* relative expression	two-way ANOVA	interaction genotype x age F(2,26) = 0.08149; p = 0.9220; age F(2,26) = 4.275; p = 0.0248; genotype F(1,26) = 0.5170; p = 0.4785	Šídák's multiple comparisons test	-
**7. CB2R deletion changes microglial morphology**	**7b:** Soma size	two-way ANOVA	interaction genotype x age F(2,1000) = 1.786; p = 0.1681; age F(2,1000) = 22.97; p < 0.0001; genotype F(1,1000) = 15.99; p < 0.0001	Šídák's multiple comparisons test	**WT vs CB2R-/- 18 months** p = 0.0006; **WT** 3-months vs 18-months p < 0.0001, 12-months vs 18-months p < 0.0001; **CB2R-/-** 3-months vs 18-months p =0.0276; 12-months vs 18-months p = 0.0029
	**7c:** Volume	two-way ANOVA	interaction genotype x age F(2,316) = 0.1823; p = 0.8334; age F(2,316) = 1.076; p = 0.3422; genotype F(1,316) = 11.6; p = 0.0007	Šídák's multiple comparisons test	**WT vs CB2R-/- 3 months** p = 0.0462
	**7d:** Ramification index	two-way ANOVA	interaction genotype x age F(2,317)= 0.4927; p = 0.6114; age F(2,317) = 1.279; p = 0.2797; genotype F(1,317) = 15.18; p = 0.0001	Šídák's multiple comparisons test	**WT vs CB2R-/- 3 months** p = 0.0101
	**7e:** Polarity index	two-way ANOVA	interaction genotype x age F2,340 = 0.09658; p = 0.9080; age F2,340 = 2.283; p = 0.1035; genotype F1,340 = 15.27; p = 0.0001	Šídák's multiple comparisons test	**WT vs CB2R-/- 3 months** p = 0.0298
	**7f:** Tree length	two-way ANOVA	interaction genotype x age F(2,329) = 1.426; p = 0.2418; age F(2,329) = 0.4238; p = 0.6549; genotype F(1,329) = 13.02; p = 0.0004	Šídák's multiple comparisons test	**WT vs CB2R-/- 3 months** p = 0.0025
	**7g:** Average branch length	two-way ANOVA	interaction genotype x age F(2,326) = 3.519; p = 0.0308; age F(2,326) = 1.295; p = 0.2754; genotype F(1,326) = 5.847; p = 0.0161	Šídák's multiple comparisons test	**WT vs CB2R-/- 18 months** p = 0.0054; **CB2R-/-** 12-months vs 18-months p =0.0187
**S1. Memory**	**S1a:** Novelty preference (1h)	one-sample t-test (hypothetical mean = 50)	**3-month WT** p= 0.0021		
	**S1a**: Novelty preference (1h)	two-way ANOVA	age x genotype F(2,77) = 2.910; p = 0.0605; age F(2,77) = 3.041; p = 0.0535	Šídák's multiple comparisons test	**WT vs CB2R-/- 18-months** p = 0.0483
	**1b**: MWM probe trial (preference for platform-associated quadrant)	one-sample t-test (hypothetical mean = 22.5)	**3-month WT** p =0.0088, **CB2R-/-** p = 0.0219; **12-month WT** p =0.0002, **CB2R-/-** p = 0.0008; **18-month WT** p =0.0421, **CB2R-/-** p = 0.0018		
**S2. Motility**	**S2a:** Distance: open-filed like test	two-way ANOVA	interaction genotype x age F(2,46) = 1.743; p = 0.1864; age F(2,46) = 0.7420; p = 0.4818; genotype F(1,46) = 0.01203; p = 0.9131		

		Statistical test		Posthoc test	
**Figure**	**Panel**	**Test name**	**result**	**Test name**	**result**
	**S2a**: Velocity: PR test	two-way ANOVA	interaction genotype x age F(2,83) = 0.2221; p = 0.8013; age F(2,83) = 1.701; p = 0.1888; genotype F(1,83) = 0.9010; p = 0.3453		
	**S2c**: Velocity: MWM test	two-way ANOVA	interaction genotype x age F(2,81) = 0.3312; p = 0.7190; age F(2,81) = 6.678; p = 0.0021; genotype F(1,81) = 1.954; p = 0.1660		**WT** 3-months vs 12-months p = 0.0083
